# Conventional and unconventional secretory proteins expressed with silkworm bombyxin signal peptide display functional fidelity

**DOI:** 10.1038/s41598-017-14833-8

**Published:** 2017-11-03

**Authors:** Sungjo Park, D. Kent Arrell, Santiago Reyes, Enoch Y. Park, Andre Terzic

**Affiliations:** 1 Center for Regenerative Medicine, Mayo Clinic, Rochester, Minnesota USA; 2 Marriott Heart Disease Research Program, Departments of Cardiovascular Medicine, Molecular Pharmacology and Experimental Therapeutics, and Medical Genetics, Mayo Clinic, Rochester, Minnesota USA; 3 Laboratory of Biotechnology, Graduate School of Science and Technology, Shizuoka University, Shizuoka, Japan; 4 Present Address: Department of Surgery, Wake Forest School of Medicine, Winston-Salem, North Carolina, USA

## Abstract

Growth factors are signaling molecules which orchestrate cell growth, proliferation and differentiation. The majority are secreted proteins, exported through the classical endoplasmic reticulum (ER)/Golgi-dependent pathway, but a few are released by unconventional ER/Golgi-independent means. Human fibroblast growth factor 2 (FGF2) and insulin-like growth factor 1 (IGF1), are canonical prototypes secreted by the unconventional and conventional pathway, respectively. We herein examined whether expression of these two growth factors in the *Bombyx mori* nucleopolyhedrovirus (BmNPV)-based silkworm expression system with its innate signal peptide, bombyxin, secures structural homogeneity at the signal peptide cleavage site regardless of the native secretory route. Proteomic analysis mapped structural microheterogeneity of signal peptide cleavage at the amino terminus of FGF2, whereas IGF1 displayed homogeneous amino-terminal cleavage with complete removal of the bombyxin signal peptide. A cell proliferation assay revealed potent functional activity of both FGF2 and IGF1, suggesting that FGF2 amino-terminal microheterogeneity does not alter mitogenic activity. These findings demonstrate that the occurrence of amino-terminal structural homogeneity may be associated with the original secretion mechanism of a particular growth factor. Furthermore, our results highlight the bombyxin signal peptide as a reliable secretion sequence applicable to mass production of functionally active secretory proteins in a silkworm-based expression platform.

## Introduction

Growth factors are essential proteins in cell signaling. Binding to specific cell surface receptors, these molecules orchestrate complex regulatory network functions including cell growth, proliferation, differentiation and maturation^1–3^. Used in combination, growth factors can prime and guide stem cells to differentiate into desired lineage-specific progeny^4,5^, underscoring their increased use in regenerative medicine applications.

Growth factors are extracellular proteins secreted by one of two different pathways in eukaryotic cells. The vast majority implement a conventional endoplasmic reticulum (ER)/Golgi-dependent pathway to gain access to the extracellular space across the plasma membrane. The presence of an amino-terminal signal peptide demarcates proteins destined for this biological route^6,7^. The nascent signal peptide emerging from the ribosome secures targeting to the ER surface, where the signal peptide is typically cleaved at a specific amino acid residue by signal peptidases before translation resumes. Newly synthesized proteins are folded into a native state within the ER and ultimately secreted. A few growth factors, however, are released in a manner independent of signal peptides and the ER/Golgi-dependent pathway, using a process collectively termed “unconventional secretion”^8,9^. Translocation of fibroblast growth factor 2 (FGF2), an essential component of human embryonic stem cell culture medium as well as a stimulant of angiogenesis, is a canonical example of unconventional secretion and, unlike classically secreted proteins, FGF2 does not require protein unfolding in order to be released into the extracellular space^8–10^.

Due to broad and intricate biological activities, growth factors are increasingly recognized as valuable in research, theranostic and biopharmaceutical applications. Different approaches at manipulating intrinsic and bioengineered signal peptides have been evaluated for optimizing heterologous expression of growth factors^11–22^. Nevertheless, structural sequence homogeneity at the signal peptide cleavage site in bioengineered growth factors secreted by the unconventional pathway is largely undocumented. Heterogeneities of therapeutic growth factors due to alternative cleavage sites of the signal peptides may cause safety concerns depending on the nature of the heterogeneity and the effects of functional activity.

Here, we examined whether the expression of growth factors in silkworm *Bombyx mori* secures structural homogeneity at the amino-terminal signal peptide cleavage site regardless of the secretory route. Utilizing the *Bombyx mori* nucleopolyhedrovirus (BmNPV)-based silkworm expression system that enables the use of bombyxin, a silkworm brain-secretory hormone, we produced recombinant FGF2 and insulin-like growth factor 1 (IGF1), recognized examples of unconventional and conventional secretory pathways, respectively. Proteomic analysis revealed structural microheterogeneity of FGF2 and homogeneity of IGF1 at their bombyxin signal peptide cleavage sites. Both bioengineered growth factors demonstrated full biological functionality indicating that amino-terminal microheterogeneity does not alter FGF2 mitogenic activity, suggesting that the amino-terminal structural homogeneity of recombinant growth factors may be associated with the nature of the growth factor’s secretion pathway. Importantly, these findings demonstrate that the silkworm-trophic bombyxin signal peptide is a reliable secretion sequence for mass production of secretory growth factors, leveraging the BmNPV-based silkworm expression system for eukaryotic recombinant secretory protein production.

## Results

### FGF2 and IGF1 production in silkworm

**Figure 1:**
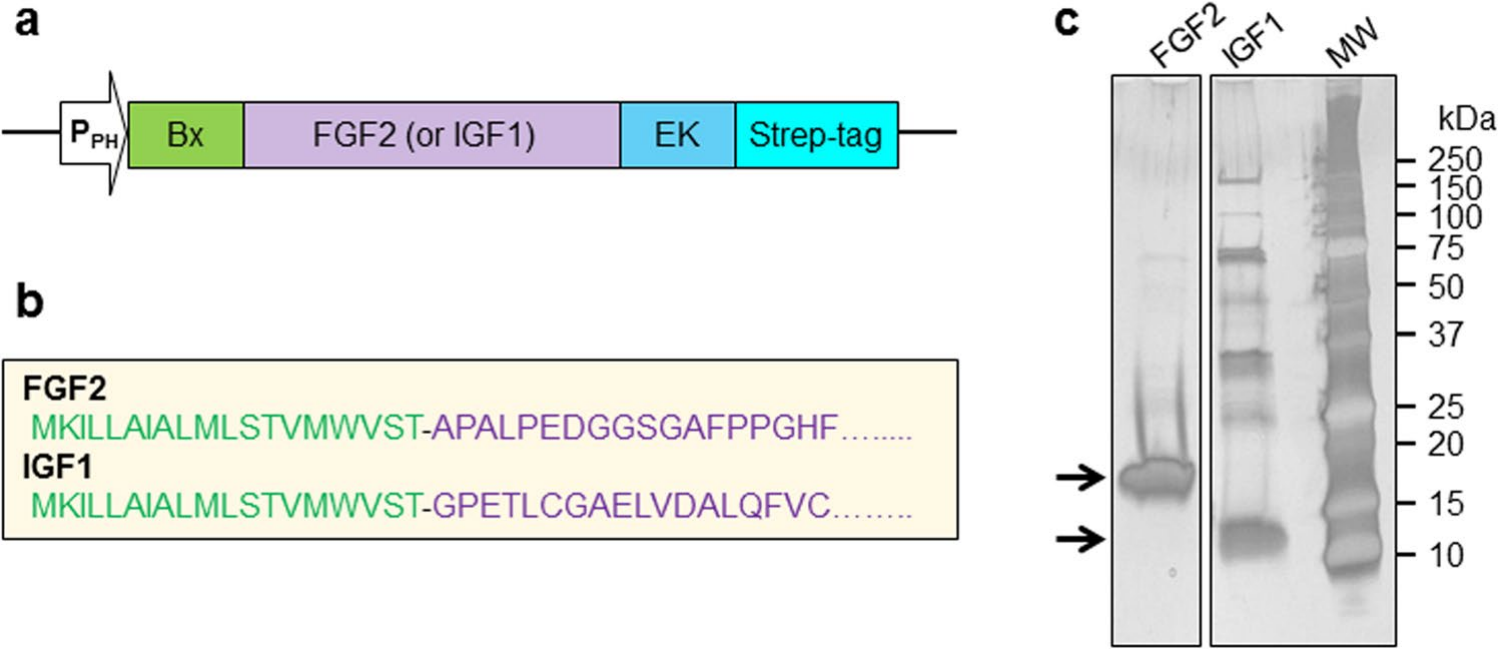
Recombinant FGF2 and IGF1 constructs and expression purification. (**a**) Human FGF2 and IGF1 were expressed in silkworm *Bombyx mori* fat body with an amino-terminal bombyxin signal peptide (Bx), a carboxy-terminal enterokinase (EK) recognition site and Strep-tag, under the control of polyhedrin promoter (P_PH_). (**b**) Bombyxin signal peptide (marked in green) is composed of 19 amino acids fused with Ala-FGF2 or IGF1. (**c**) Aliquots of recombinant FGF2 and IGF1 purified by Strep-Tactin affinity purification were resolved by SDS-PAGE and evaluated for purity by silver staining.

For expression of mature proteins, FGF2 and IGF1 were engineered with an amino-terminal bombyxin signal peptide, a carboxy-terminal enterokinase cleavage site and Strep-tag (Fig. 1a,b and Supplementary Fig. 1). Without an amino-terminal bombyxin signal peptide, the expression of these growth factors was not detected (not shown), indicating its necessity in securing production of secreted recombinant protein from the silkworm-based expression system. Recombinant FGF2 and IGF1 from silkworm fat body were purified using a Strep-Tactin affinity gel column. After extensively washing the column, growth factors were eluted with buffer containing desthiobiotin. Silver stained SDS-PAGE indicated the main purification components migrated at the anticipated molecular masses for FGF2 and IGF1, with estimated recombinant protein purity of ~95% and ~70%, respectively (Fig. 1c). Due to its acidic nature (pI = 5.5), IGF1 migrates slightly higher than the expected molecular weight (Fig. 1c). Recombinant FGF2 and IGF1 were also extracted from hemolymph, but the expression was limited (not shown) precluding further evaluation from this source. Fusion to the bombyxin signal peptide was required for and enabled expression of the growth factors in a silkworm bioreactor, and one-step purification delivered enriched proteins, ready for proteomic and functional analysis.

### Proteomic analysis of silkworm expressed FGF2

**Figure 2:**
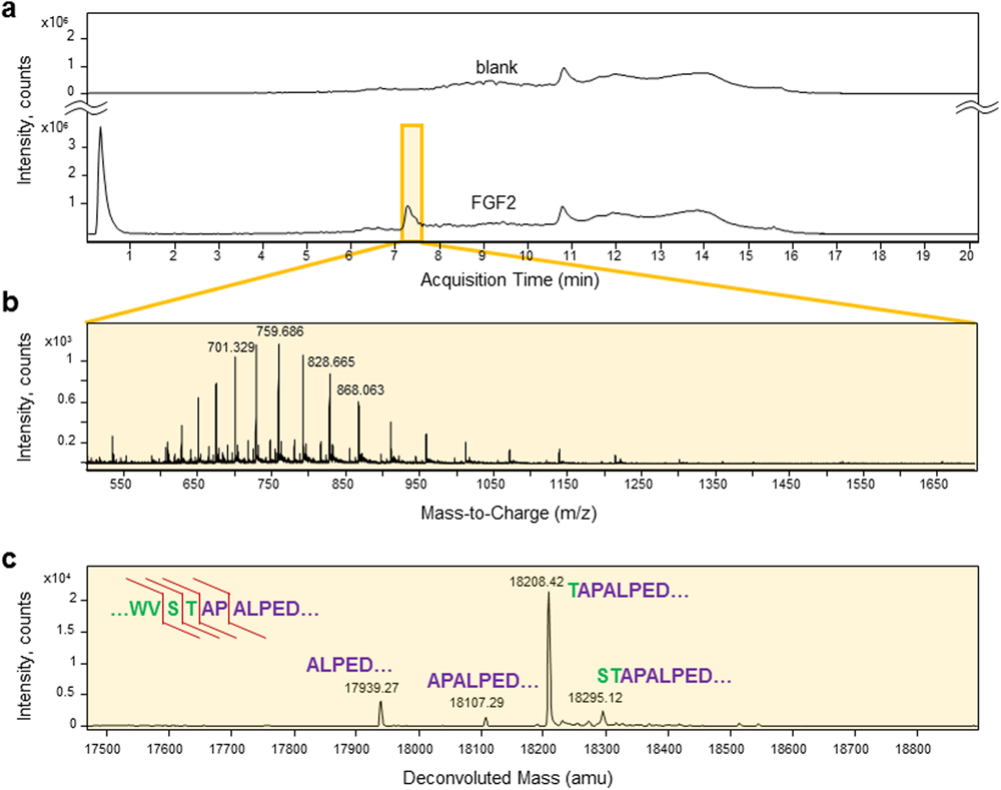
LC-MS analysis reveals signal peptide cleavage heterogeneity of recombinant FGF2 expressed with an amino-terminal bombyxin signal peptide. (**a**) The total ion chromatogram of FGF2 compared with a negative control run revealed a characteristic peak at ~7.2 min. (**b**) The proteomic spectrum corresponding to the peak at 7.1–7.6 min with 29 averaged scans displayed several characteristic mass-to-charge ratio peaks. (**c**) The transformed, deconvoluted mass spectrum of recombinant FGF2 exhibited four distinctive molecular masses, each corresponding to a different cleavage site near the junction of the bombyxin signal peptide and mature FGF2. Detected cleavage sites are indicated by red lines in the upper left sequence of the signal peptide – FGF2 junction.

The total ion chromatogram of recombinant FGF2 compared with the negative control run revealed a characteristic peak eluting at ~7.2 min, which displayed several mass-to-charge ratio peaks (Fig. 2a and b). Precise bombyxin signal peptide cleavage should result in FGF2 having a final mass of 18107.29 amu. The deconvoluted FGF2 mass spectrum, however, exhibited four distinct molecular masses, representing different cleavage sites yielding FGF2 beginning at positions ALPED… (MW = 17939.27 amu), APALPED… (MW = 18107.29 amu), TAPALPED… (MW = 18208.42 amu), and STAPALPED…. (MW = 18295.12 amu) (Fig. 2c and Table 1). The major peak at 18208.42 represents about 73% of the four heterogeneous forms, indicating that the majority of recombinant FGF2 contains one remaining threonine residue from the bombyxin signal peptide, whereas the anticipated cleavage to yield precise full sequence FGF2 represented only 5% of detected forms. Bioinformatics prediction of this signal peptide cleavage site by SignalP 4.1 (http://www.cbs.dtu.dk/services/SignalP/), a purely neural network-based method^23^, predicts complete removal of the bombyxin signal peptide. In contrast to IGF1, FGF2 is secreted *in vivo* through the unconventional pathway, which does not require protein unfolding in order to be released into the extracellular space^8–10^. This could restrict signal peptide recognition site exposure to endogenous peptidases, leading to incomplete cleavage of the signal peptide. The observed structural microheterogeneity of the amino-terminal cleavages in recombinant FGF2 derived from silkworm may be a distinct feature of unconventional secretory proteins.

**Table 1:** Characteristics of FGF2 and IGF1 molecular masses detected by mass spectrometry.

Growth factor	Observed mass, non-reduced form	Observed mass, reduced form	Cleavage site	Comments
FGF2	18295.1		↓ST-APALPED…..	
	18208.4		↓T-APALPED….	
	18107.3		↓APALPED….	Mature Ala-FGF2
	17939.3		↓ALPED….	
IGF1	9277.6	9283.4	↓GPETLC….	Mature IGF1
		9186.8	↓GPETLC….	IGF1 (K102 truncation) methionine sulfone
	9166.3	9171.9	↓GPETLC….	IGF1 (K102 truncation) methionine sulfoxide
	9149.3	9155.2	↓GPETLC….	IGF1 (K102 truncation)
		9139.3		Impurity
	9123.0	9123.2		Impurity
	9010.9			Impurity

### Proteomic analysis of silkworm expressed IGF1

**Figure 3:**
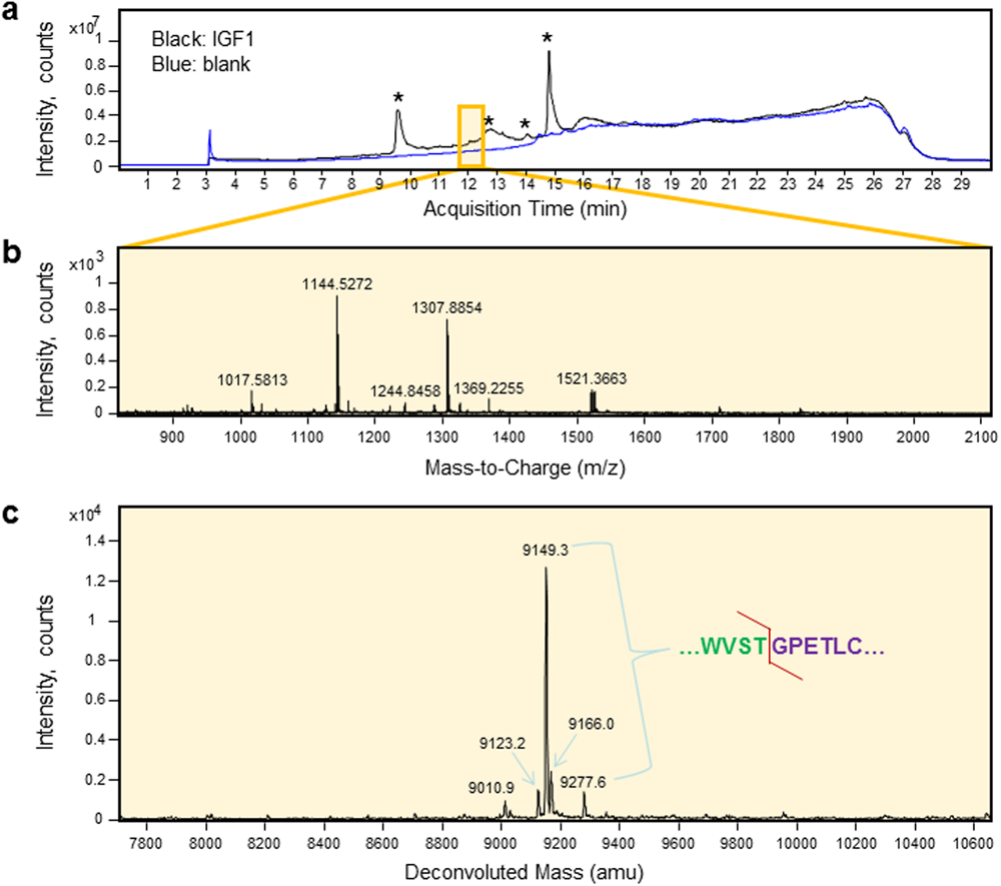
LC-MS analysis reveals signal peptide cleavage homogeneity of recombinant IGF1 expressed with an amino-terminal bombyxin signal peptide. (**a**) The total ion chromatogram of IGF1 compared with a negative control run does not reveal any prominent IGF1 peak due to the high intensity of single charged background ions representing masses <1 kDa, which are marked by asterisks. (**b**) The mass spectrum corresponding to the chromatogram at 11.8–12.3 min with 33 averaged scans displayed several characteristic mass-to-charge ratio peaks. (**c**) The transformed, deconvoluted mass spectrum of recombinant IGF1 displayed peaks at 9149.3, 9166.3, and 9277.6 amu, which correspond to molecular masses of IGF1 with different structures but homogeneous cleavage between the signal peptide and IGF1 starting position at GPETLC. Minor peaks that do not match molecular masses of any cleavage forms of IGF1 could be contaminants detected in purified IGF1 (see Fig. 1c). Detected cleavage site is indicated by a red line in the upper right sequence of the signal peptide – IGF1 junction.

Conventional secretory protein IGF1 possesses three internal disulfide bonds, so proteomic analysis was performed in the absence and presence of a reducing agent, tris(2-carboxyethyl)phosphine (TCEP). The total ion chromatogram of recombinant non-reduced IGF1 did not display a distinctive IGF1 peak due to intense peaks arising from the presence of singly charged background ions with masses less than 1 kDa, but an average of 33 scans at ~12 min revealed several mass-to-charge spikes and a deconvoluted mass spectrum corresponding to IGF1 (Fig. 3). Precise bombyxin signal peptide cleavage in non-reduced IGF1 should produce a final mass of 9277.6 amu. Of note, the deconvoluted mass spectrum revealed several unique peaks. The three peaks at 9149.3, 9166.0 and 9277.6 amu represent different structures of IGF1 due to methionine oxidation or truncation of C-terminal lysine, but all with homogeneous signal peptide cleave starting at IGF1 position GPETLC… (Fig. 3c and Table 1). Minor peaks at 9010.9 and 9123 amu did not match any forms of IGF1 resulting from an altered cleavage site, and it is possible that these peaks arose from contaminants remaining following protein purification.

**Figure 4:**
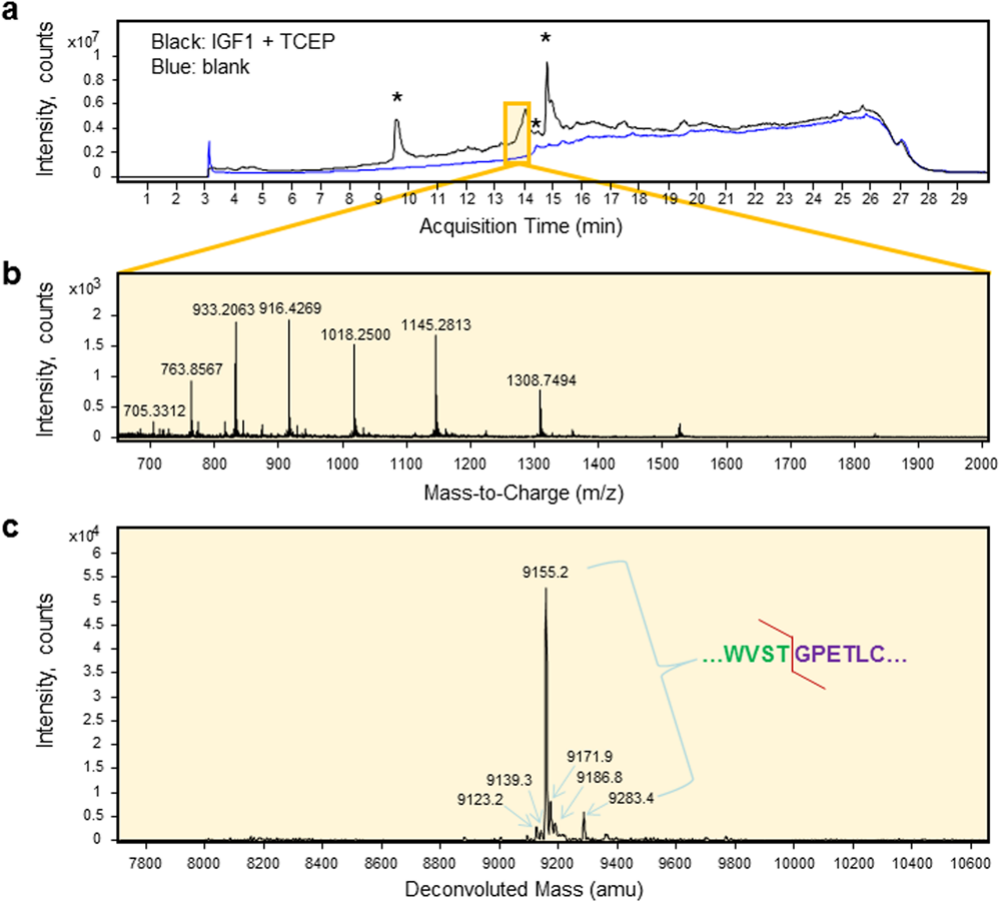
LC-MS analysis reveals signal peptide cleavage homogeneity of recombinant IGF1 expressed with an amino-terminal bombyxin signal peptide in the presence of reducing agent TCEP. (**a**) Reduced IGF1 eluted later than the non-reduced form in the total ion chromatogram. Single charged background ions with high intensity representing masses < 1 kDa are marked with asterisks. (**b**) The proteomic spectrum corresponding to the peak at 13.6–14.1 min with 27 averaged scans displayed several characteristic mass-to-charge ratio peaks. (**c**) The transformed, deconvoluted mass spectrum of recombinant reduced IGF1 displayed several peaks at 9155.2, 9171.9, 9186.8 and 9283.4 amu representing molecular masses of IGF1 with different structures. Minor peaks that do not match molecular masses of any cleavage forms of IGF1 could represent contaminants detected in purified IGF1 (see Fig. 1c). Detected cleavage site is indicated by a red line in the upper right sequence of the signal peptide – IGF1 junction.

Following reduction with TCEP, elution of IGF1 shifted to ~14 min in the total ion chromatogram, which produced characteristic mass-to-charge peaks and the deconvoluted mass spectrum of reduced IGF1 (Fig. 4 and Table 1). Peaks at 9149.3, 1966.3 and 9277.6 amu in non-reduced IGF1 moved to 9155.2, 9171.9 and 9283.4 amu in reduced form, respectively, indicating a 6 Da increase due to the reduction of three internal disulfide bridges. These deconvoluted masses match the molecular mass of IGF1 with different structures but identical signal peptide cleavage. Minor peaks at 9123.2 and 9139.3 amu did not match the molecular masses of any cleavage forms of IGF1 and did not correspond to any 6 Da shifts following TCEP reduction, indicating that these were attributable to non-IGF1 impurities. Consistent with experimental findings, SignalP 4.1 predicted complete removal of the bombyxin signal peptide from IGF1. Therefore, the bombyxin signal peptide of IGF1 secreted by conventional ER/Golgi-dependent pathway was readily recognized by the signal peptidase, producing homogeneous cleavage at the amino-terminus of IGF1.

### Biological activity of IGF1 and FGF2

**Figure 5:**
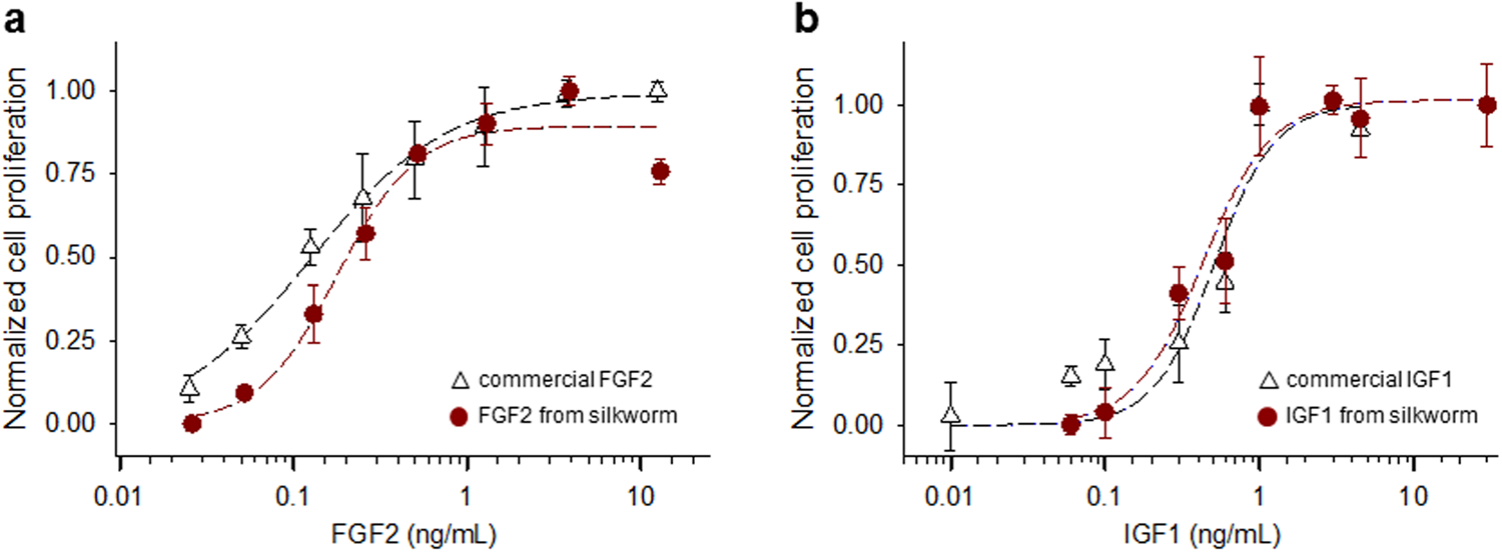
Comparable cell proliferation activity of recombinant and commercially available FGF2 and IGF1. Cell proliferation activities were normalized after 48 hours of incubation with the addition of various concentrations of recombinant and commercially available FGF2 (**a**) or IGF1 (**b**). Cells without growth factor treatments were used as a reference. Data points are averages of 4 independent measurements ± standard error, and curves are Hill plots constructed based on fitting results.

To evaluate biological activity of recombinant FGF2 expressed in silkworm, mitogenic experiments were performed using NIH 3T3 cells. The rate of cell proliferation in the presence of FGF2 after 48 h of culture was greater than in control samples without FGF2, and displayed dose-dependence with ED_50_ = 0.18 ± 0.03 ng/mL (n = 4; Fig. 5a). Although recombinant FGF2 demonstrated structural heterogeneity at the amino-terminal end following bombyxin signal peptide cleavage, the ED_50_ obtained here is comparable to values acquired with commercially available FGF2, with ED_50_ = 0.13 ± 0.02 ng/mL (n = 4; Fig. 5a) and consistent with published results^17,18,21^, suggesting that amino-terminal structural heterogeneity does not affect FGF2 functional activity. Similarly, biological activity of silkworm expressed IGF1 was also analyzed after 48 h incubation at various recombinant IGF1 concentrations. Dose-dependent cell proliferation results with IGF1 revealed an ED_50_ = 0.43 ± 0.07 ng/mL (n = 4; Fig. 5b), again comparable to values acquired using commercially available IGF1, with ED_50_ = 0.51 ± 0.15 ng/mL (n = 4; Fig. 5b) and in line with established values^24,25^. Collectively, both conventionally and unconventionally secreted growth factors expressed in silkworm and secreted here via the bombyxin secretion signal peptide retain potent functionality to stimulate cell proliferation.

## Discussion

Growth factors are secretory signaling molecules that mediate vital cellular processes^1–3^. Accordingly, they are increasingly recognized as valuable tools in research and biopharmaceutical development with a myriad of established and potential applications. However, the need for precise functional activity requires high fidelity and structural homogeneity of bioengineered recombinant growth factors. Utilizing proteomic analysis, we here revealed amino-terminal structural heterogeneity of FGF2 and homogeneity of IGF1 when expressed with an endogenous bombyxin signal peptide in the BmNPV-based silkworm expression system. Therefore, amino-terminal structural fidelity differs between the two growth factors, which may be associated with their original secretion mechanism. Importantly, as demonstrated here bombyxin signal peptide offers a reliable secretion sequence applicable to large-scale production of active biological forms of growth factors generated in the silkworm-based expression system.

Human FGF2 lacks a conventional secretion signal sequence, but is known to employ an unconventional ER/Golgi-independent secretion pathway for release into the extracellular space^2,8^. Proteolytic processing at the amino-terminal end delivers multiple forms of authentic FGF2 in eukaryotic cells and the yeast *Saccharomyces cerevisiae* ^26,27^. Contrary to native FGF2 with multiple forms, heterologous expression of FGF2 with the amino-terminal bombyxin signal peptide would be expected to remove the signal peptide by signal peptidase *in vivo*, ultimately generating a homogeneous cleavage site as observed in recombinant IGF1 production. Bioinformatic analysis of cleavage sites predicted by SignalP supported this notion. Detection of four different cleavage sites of recombinant FGF2, however, suggests that the signal peptide in FGF2 may not allow for forced secretion along the conventional ER/Golgi-associated pathway in the silkworm expression system.

The molecular mechanism of unconventional secretion has been enigmatic although several examples were observed in eukaryotes as well as lower organisms including *Dictyostelium* ^8,9,28–30^. Emerging studies revealed that translocation of FGF2 across plasma membranes comprises several processes: (1) recruitment of fully folded FGF2 at the inner leaflet of the plasma membrane mediated by phosphatidylinositol 4,5-bisphosphate, where Tec kinase induces phosphorylation of FGF2, (2) oligomerization of FGF2 leading to membrane insertion and pore formation, and (3) extracellular trapping of dissociated monomeric FGF2 facilitated by membrane-proximal heparan sulfate proteoglycans^8–10,31^. As FGF2 secretion via the unconventional pathway is rigorously dependent on its folding, the multiple processes of FGF2 translocation across the plasma membrane may contribute to stringent quality control underscoring the biological and functional significance of FGF2.

Bombyxin is a prothoracicotropic hormone (brain-secretory hormone) of silkworm, which functions to reduce the concentration of trehalose, the major blood sugar of silkworm, and stimulates prothoracic glands to release ecdysone, the insect molting hormone^32,33^. Although bombyxin derives from invertebrates, it is a member of the insulin family with sequence as well as structural similarity with insulin family peptides such as human relaxin, insulin and insulin-like growth factor^34,35^. Composed of 19 amino acids, bombyxin signal peptide, like other canonical signal peptides, displays very little sequence conservation. However, bombyxin signal peptide harbors three common structural features: an amino-terminal region with a net positive charge; a central hydrophobic core region; and a carboxy-terminal region with neutral but polar residues containing the cleavage site, which is processed by signal peptidases^36,37^. All of these features are essential for the secretory function of signal peptides.

Bombyxin signal peptide was previously utilized to target non-secretory proteins, such as urease subunit B and β1,3-N-acetylglucosamyltransferase 2, for export via the secretion pathway of an insect expression system^14,15,22^. Secretory expression improves protein production yield, underscoring the strategy of using the bombyxin signal peptide as effective in a large-scale protein production platform^22^. Secreted proteins may be extracted from either silkworm hemolymph or fat body. Our previous studies have shown that recombinant proteins more readily express in the fat body of silkworm than in the hemolymph when cysteine protease deficient BmNPV bacmid is used^14^. More than 93% of FGF2 secreted via the baculovirus major envelope glycoprotein (gp67) signal sequence was present in the silkworm fat body^17^, demonstrating greater yield compared to the hemolymph. Consistent with these findings, we herein showed that recombinant FGF2 and IGF1, when produced with cysteine protease deficient BmNPV bacmid, were also mainly expressed in the silkworm fat body indicating a production predilection.

In summary, FGF2 and IGF1 fused with bombyxin signal peptide were successfully produced using the BmNPV-based silkworm expression system. Heterologous FGF2 displays structural microheterogeneity whereas recombinant IGF1 reveals homogeneity at the amino-terminal end, underscoring that structural integrity at the amino terminus of recombinant growth factors, even in the presence of an amino-terminal signal peptide, may be closely associated with the secretory mechanism. Both recombinant FGF2 and IGF1 demonstrate potent functional activity to stimulate cell proliferation. Thus, the structural fidelity of FGF2 and IGF1 provides mechanistic insights into the secretory mechanism of growth factors in the heterologous silkworm expression system. Collectively, the present study reveals that the bombyxin signal peptide offers a reliable secretion sequence applicable for large-scale production of active biological forms of growth factors normally secreted by either conventional or unconventional pathways.

## Methods

### Construction of recombinant human FGF2 and IGF1

The complementary DNA (cDNA) fragments of human basic fibroblast growth factor (FGF2: Ala-Pro143-Ser288; UniProt accession: P09038) or human insulin-like growth factor 1 (IGF1: Gly49-Ala118; UniProt accession: P05019), were cloned along with an amino-terminal bombyxin signal peptide (MKILLAIALMLSTVMWVST), a carboxy-terminal enterokinase-specific cleavage site and Strep-tag into a pFastbac1 (Invitrogen) expression vector (Fig. 1 and Supplementary Fig. S1). Alanine was introduced at the N-terminus of FGF2 to avoid proline-induced disruption of α-helix secondary structure of the bombyxin signal peptide. Constructs confirmed by DNA sequencing were transformed into *Escherichia coli Bm* DH10Bac-CP^-^ competent cells containing the cysteine protease-deficient BmNPV bacmid, following the standard protocol of Bac-to-Bac Baculovirus Expression System (Invitrogen). Recombinant BmNPV bacmid containing FGF2 or IGF1 was purified, and the identity of recombinant bacmids was confirmed^38^.

### Expression and purification of recombinant human FGF2 and IGF1 in silkworm

Recombinant BmNPV bacmid was injected into the dorsum of silkworm larvae with DMRIE-C reagent (Invitrogen). Bacmid injected larvae were reared at room temperature for 6–7 days, and then the fat body was collected by dissection, and immediately frozen at −70 °C until further analysis. Protein purification was carried out at 4 °C to minimize aggregation and protease activity^38–40^. Larval fat bodies were homogenized in 10 mL of lysis buffer (50 mM sodium phosphate, 300 mM NaCl, pH 7.5 and 0.1% Triton X-100) containing an EDTA-free protease inhibitor tablet (Roche) using a Dounce tissue grinder. Cell debris was discarded by pelleting through centrifugation at 12,000 *g* for 30 min. After filtration using a 0.45 μm syringe filter, the supernatant was loaded onto a 1 mL Strep-Tactin resin affinity column (Qiagen), pre-equilibrated with lysis buffer. The column was extensively washed with lysis buffer and eluted with lysis buffer containing 2.5 mM desthiobiotin (Sigma). The concentration of purified growth factors was quantified using an extinction coefficient of 21680 (Mcm)^−1^ for FGF2 and 10345 (Mcm)^−1^ for IGF1 at 280 nm. Purity and integrity of FGF2 and IGF1 were confirmed by silver staining following protein resolution by SDS-PAGE.

### Proteomic analysis

Enriched FGF2 and IGF1 samples were analyzed at the Mayo Proteomics Core Facility using liquid chromatography separation combined with a time-of-flight mass spectrometry (LC-TOF-MS). Injections of 8 µL of recombinant protein solution (20 ng/µL) were loaded onto an Agilent Poroshell 300SB-C8 column (1 × 75 mm, 5 µm) or Zorbax SB-C8 column (2.1 × 50 mm, 3.5 µm) on an Agilent 1100 LC system. Separation was achieved using a linear gradient of H_2_O, acetonitrile, and 0.1% formic acid. Mass spectrometry was performed on an Agilent MSD/TOF system using electrospray ionization in positive ion mode. The mass spectrometry acquisition method was set to acquire data over the entire duration of each LC gradient, in the mass range from 400 to 2500 *m/z*. Chromatograms and raw spectra were produced by Agilent Qualitative Analysis Software. Deconvoluted spectra of molecular mass were generated from Agilent Protein BioConfirm Software.

### Cell proliferation assay

The biological activity of recombinant growth factors was measured using CyQUANT Cell Proliferation Assay Kit (Invitrogen). This assay kit determines the density of cells in culture using a green fluorescent dye, CyQUANT GR, which binds to cellular nucleic acids from lysed cells. Mouse embryo fibroblast cells (NIH/3T3, ATCC#CRL-1658) were prepared in the wells of a microplate containing growth factors ranging from 0 to 30 ng/mL concentrations. After incubation of cells at 37 °C for up to three days, the medium was removed from the wells and stored at −70 °C until further analysis. Commercially available FGF2 and IGF1 (R&D Systems) were used as positive controls for the cell proliferation assay. To quantitate cell proliferation, frozen plates were thawed at room temperature, and 200 µL of the CyQUANT GR dye/cell-lysis buffer was added. Fluorescence was measured with excitation at 485 nm and emission at 520 nm using a FLUOstar Omega microplate reader (BMG Labtech). Data points are averages of 4 independent measurements ± standard error. Curves were constructed based on fitting of normalized fluorescent data to Hill’s equation using SigmaPlot (Systat Software).

## Electronic supplementary material

Supplementary Figure S1
