# Supplementary material for: Conventional and unconventional secretory proteins expressed with silkworm bombyxin signal peptide display functional fidelity
Source: Sci Rep. 2017 Nov 3;7:14499. doi: 10.1038/s41598-017-14833-8 (PMC5670176; doi:10.1038/s41598-017-14833-8)
Supplement: Supplementary file 1 — Supplementary Figure S1 [file 41598_2017_14833_MOESM1_ESM.pdf]

Supporting information

Conventional and unconventional secretory proteins expressed with silkworm bombyxin signal peptide display functional fidelity

Sungjo Park[1,2], D. Kent Arrell[1,2], Santiago Reyes[1,2],

Enoch Y Park[3] and Andre Terzic[1,2]

[1]Center for Regenerative Medicine, Mayo Clinic, Rochester, Minnesota, USA

[2]Marriott Heart Disease Research Program, Departments of Cardiovascular Diseases, Medicine, Molecular Pharmacology and Experimental Therapeutics, and Medical Genetics, Mayo Clinic, Rochester, Minnesota, USA

[3]Laboratory of Biotechnology, Graduate School of Science and Technology, Shizuoka University, Shizuoka, Japan

*Corresponding author: A. Terzic, Mayo Clinic, 200 First Street SW, Rochester, MN 55905, USA; Tel: +1 507-284-5514; Fax: +1 507-266-9936; E-mail: terzic.andre@mayo.edu

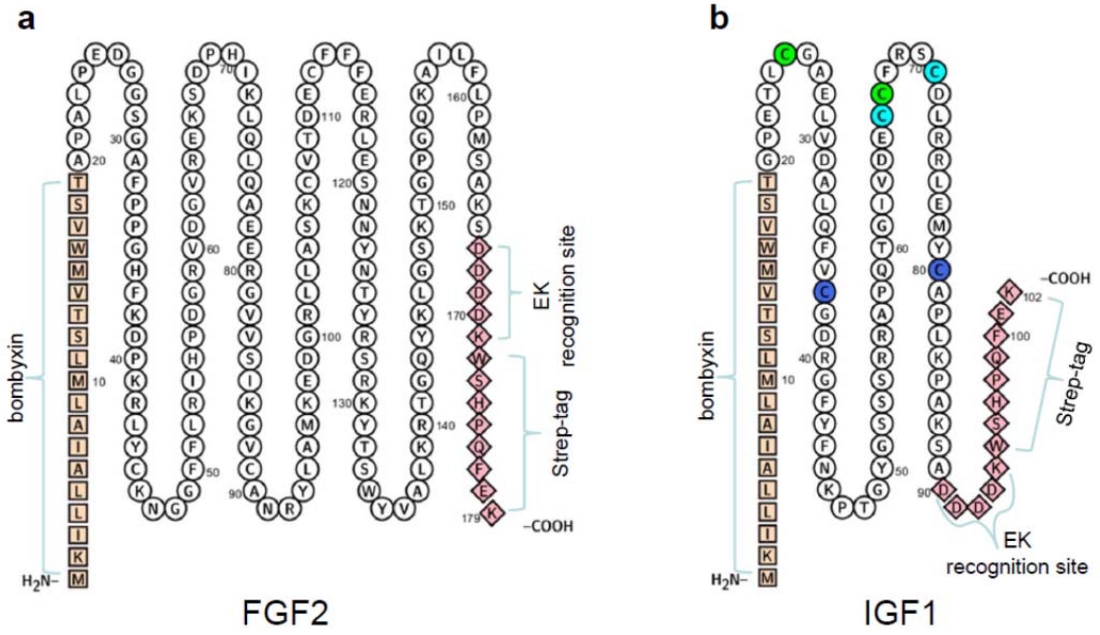

Supplementary Figure S1. Amino acid sequences of human Ala-FGF2 (UniProt accession: P09038) and IGF1 (UniProt accession: P05019) constructs containing an amino-terminal bombyxin signal peptide, a carboxy-terminal enterokinase (EK) cleavage site and Strep-tag. To avoid abrupt conformational change introduced by the N-terminal proline of FGF2 near the junction between bombyxin signal peptide and mature FGF2, an alanine residue (Ala at position 20) was added to the N-terminus of FGF2. Cysteine residues forming disulfide bonds in IGF1 are colored with disulfide pairs in green, cyan and blue. Illustrations of primary sequences were generated using Protter (http://wlab.ethz.ch/protter/start/).
